# Asymptomatic malaria, growth status, and anaemia among children in Lao People’s Democratic Republic: a cross-sectional study

**DOI:** 10.1186/s12936-016-1548-3

**Published:** 2016-10-18

**Authors:** Takeshi Akiyama, Tiengkham Pongvongsa, Souraxay Phrommala, Tomoyo Taniguchi, Yuba Inamine, Rie Takeuchi, Tadashi Watanabe, Futoshi Nishimoto, Kazuhiko Moji, Shigeyuki Kano, Hisami Watanabe, Jun Kobayashi

**Affiliations:** 1 Center of Molecular Bioscineces, Tropical Biosphere Research Center, University of the Ryukyus, Senbaru 1, Nishihara, Okinawa Japan; 2 Nagano College of Nursing, Komagane, Nagano Japan; 3 Station of Malariology, Parasitology and Entomology, Savannakhet Health Department, Savannakhet, Lao People’s Democratic Republic; 4 National Institute of Public Health, Ministry of Health, Vientiane Capital, Lao People’s Democratic Republic; 5 Center for Medical Education, Graduate School of Medicine, Gunma University, Maebashi, Gunma Japan; 6 Institute of Tropical Medicine, Nagasaki University, Nagasaki, Nagasaki Japan; 7 Department of Cell Biology, Kyoto Pharmaceutical University, Kyoto, Japan; 8 School of Tropical Medicine and Global Health, Nagasaki University, Nagasaki, Nagasaki Japan; 9 Research Institute, National Center for Global Health and Medicine, Tokyo, Japan; 10 Graduate School of Medical and Dental Sciences, Niigata University, Niigata, Niigata Japan; 11 Graduate School of Medicine, University of the Ryukyus, Nishihara, Okinawa Japan

**Keywords:** Asymptomatic malaria, Anemia, *Plasmodium falciparum*, Malnutrition, Stunting, Underweight, Lao PDR

## Abstract

**Background:**

Asymptomatic malaria can be observed in both stable endemic areas and unstable transmission areas. However, although much attention has been given to acute malaria infections, relatively little attention has been paid to asymptomatic malaria. Nonetheless, because the asymptomatic host serves as a reservoir for the malaria parasite, asymptomatic malaria is now recognized as an important obstacle to malaria elimination. Asymptomatic malaria is also associated with anaemia, a global public health problem with serious consequences on human health as well as social and economic development. In Lao People’s Democratic Republic (Lao PDR), malaria, anaemia, and malnutrition are serious public health concerns. However, few studies have focused on the relationship between these variables. Therefore, this study investigated the relationship between asymptomatic malaria, growth status, and the prevalence of anaemia among children aged 120 months old or younger in rural villages in Lao PDR.

**Methods:**

In December 2010 and March 2011, data were collected from five villages in Savannakhet province. Anthropometric measurements, blood samples, and malaria rapid diagnostic tests were conducted. The presence of malaria was confirmed with polymerase chain reaction assays for *Plasmodium falciparum*. Underweight status, stunting, and anaemia were defined according to World Health Organization standards.

**Results:**

The mean age of participants (n = 319) was 88.3 months old (Standard Deviation: 20.6, ranged from 30–119 months old), and 20 participants (6.3 %) had an asymptomatic malaria infection, 92 (28.8 %) were anaemic, 123 (38.6 %) were underweight, and 137 (42.9 %) were stunted. Stunted children were more likely to be infected with asymptomatic malaria [odds ratio (OR) 3.34, 95 % confidence interval (CI) 1.25–8.93], and asymptomatic malaria was associated with anaemia [OR 5.17, 95 % CI 1.99–13.43].

**Conclusions:**

These results suggest a significant association between asymptomatic malaria and anaemia in children. Furthermore, stunted children were more likely to have lower Hb levels and to be infected with asymptomatic malaria than children without stunting. However, further studies examining the impact of asymptomatic malaria infection on children’s nutritional and development status are necessary.

## Background

Asymptomatic malaria can be observed in both stable endemic areas and unstable transmission areas [1–5]. Although it is difficult to define asymptomatic malaria because of a lack of standard diagnostic criteria, the most widely used criteria include the presence of parasites in peripheral thick blood smears, an axillary temperature below 37.5 °C, and the absence of malaria-related symptoms [6]. For an individual, an asymptomatic infection might be benefit for the host rather than no infection at all in order to keep the immunity against malaria [7–12]. However, for communities, asymptomatic hosts serve as a reservoir for the malaria parasite and, therefore, asymptomatic malaria is recognized as an important obstacle to malaria elimination [6].

Asymptomatic malaria has received less attention than symptomatic malaria in major studies [6]. However, some studies have also shown that asymptomatic malaria is associated with anaemia [3, 5, 13–19], a global public health problem with serious consequences for human health, as well as social and economic development [20]. Therefore, because asymptomatic malaria infections can last up to one year [21–23], they may seriously affect the host [24, 25] by causing an iron deficiency.

Studies show inconsistent results regarding the relationship between asymptomatic malaria and the growth status of children. For example, some studies have suggested that indicator of malnutrition such as stunting or underweight was associated with asymptomatic malaria, while others have not [19, 26–34]. Therefore, examining asymptomatic malaria and its association with malnutrition would be important for public health in areas where malaria coexist [27].

Anaemia, malnutrition, and malaria are all serious public health problems throughout Lao People’s Democratic Republic (Lao PDR) [20, 33] and particularly in the Savannakhet province located in the southeastern part of the country. In 2008, this province ranked third among all provinces in Lao PDR for the overall incidence of confirmed *Plasmodium falciparum* (11.5 per 1000 persons) [35]. Furthermore, wasting (low weight for height) of children in this province was benchmarked as “serious” by the World Food Programme [36].

This study aimed to investigate the relationship between asymptomatic malaria, growth status, and the prevalence of anaemia among children aged 120 months or younger in rural villages in the Savannakhet province in Lao PDR.

## Methods

### Study site

A cross-sectional survey was conducted in five villages in the Savannakhet province: Dong Savanh, Arai Yai, Kalouk Kao, and Kalouk Mai in Seponne area, December 2010 and Lahanam in March 2011. In Lao PDR, both May and December are of dry season. Malaria transmission is high rainy season between May and October [37], but transmission is perennial [38].

### Data collection

As a part of survey for active case detection, all villagers including adults were invited to the data collection in each village where interviews were conducted by a medical doctor, and body temperature, anthropometric measurements, and blood samples were taken by nurses. A rapid diagnostic test for malaria was also conducted, and positive participants were treated with artemisinin-based combination therapy according to country protocols. In the Lahanam area, age in months was calculated from the registered date of birth. In Seponne, participants’ registered date of birth was not collected from the local administration office because of coordination problems. Then children aged less than one year old were recorded their age in month from the interview of caregiver. As the caregivers did not know the exact birth date of their children, children aged one year old or older were recorded using self-reported age in completed years. In order to calculate height for age and weight for age Z-score, the age in months were estimated by adding six months to age in completed years in order to minimize the difference from real age in months of each child.

Informed consent was obtained from the principal caregiver. The National Institute of Public Health, Ministry of Health, Lao PDR approved this study in September 2008.

### Definition of asymptomatic malaria, growth status, anaemia

Asymptomatic malaria was defined as being free of fever (body temperature ≤37.5 °C) [39] and presence of malaria parasite infection and absence of any malaria related symptom at the time of data collection [40]. Malaria infection was confirmed by polymerase chain reaction (PCR) assays for *P. falciparum* in the laboratory of the University of the Ryukyus. PCR assays were conducted with the all blood samples from the eligible children regardless of the results from RDT, including negative ones. Being underweight was defined as having a weight-for-age z score ≤−2 standard deviations (SD) below the World Health Organization’s (WHO) growth standards [41]. Stunting was defined as having a height-for-age z score ≤−2 SD. Anaemia was determined by haemoglobin (Hb) levels using the HemoCue system^®^ (HemoCue^®^ AB, Ängelholm, Sweden) with a threshold of 110 g/L for children aged <5 and 115 g/L for children aged ≥5 [42, 43]. Anemia was categorized as severe, moderate and mild [43].

### Analysis

A total of 784 villagers with all ages (range: 0–70 years old) participated in anthropometric measurements, blood sample collection, and malaria rapid diagnostic tests as a part of active case detection survey. In order to use the WHO growth standards for weight-by-age, children and adults aged older than 120 months were excluded from analysis (n = 427). Children younger than 24 months of age were excluded from anthropometric measurement due to time and equipment constrains to measure their length correctly, and take blood sample (n = 7). As a consequence, the data from children aged 24 months or older, and 120 months or younger (n = 350) were included in the analysis. Among those 350 cases, data with missing data for the anthropometric or body temperature measurements, or blood collection were excluded from the analysis (n = 26).

Five symptomatic malaria cases were also excluded. Thus, 319 children were included in the final analysis. As mentioned above, the age in months among children in Seponne area were estimated by adding 6 months to age in completed years (n = 145, 45.5 %).

The demographic characteristics of the participants, rates of asymptomatic malaria infection, Hb levels, the prevalence of anaemia, weight, and height were examined. T-tests were used to compare the differences in the mean Hb level between boys/girls, underweight/not underweight, and stunting/no stunting. Odds ratios (OR) with 95 % confidence intervals (CI) stratified by growth status were used to examine the association between anaemia and asymptomatic malaria. A multivariate linear regression analysis with forced entry was conducted using Hb level as the dependent variable. The participants’ age, asymptomatic malaria infection status, and growth status (stunting/underweight) were included as independent variables. To avoid multicollinearity, height and weight were input separately, and two multivariate regression models were developed: one including weight and the other including height. The significance level for statistical testing was set at p < 0.05. IBM SPSS ver. 21 was used for statistical analysis.

## Results

### Characteristics of the participants

The mean age of participants (n = 319) was 88.3 months old (SD: 20.6) and ranged from 30–119 months old (Table 1). The proportion of male to female participants was approximately the same (boys = 50.2 % and girls = 49.8 %). The mean Hb level was 119.0 g/L (SD: 12.6). A total of 92 participants were classified as anaemic, and asymptomatic malaria infection was found in 20 participants. Among anaemic children, few cases of severe anaemia were found (n = 2), where almost children were mild or moderate anaemia. There was no severe anaemia case in the participants infected with asymptomatic malaria. Stunted children were more likely to be infected with asymptomatic malaria (OR 3.34, 95 % CI 1.25–8.93) and there were no significant differences in growth status or the prevalence of anaemia or asymptomatic malaria between male and female participants.

**Table 1:** Characteristics of the participants (n = 319)

Characteristics	N	(%)	Mean	SD	Asymptomatic *Pf* infection N	OR (95 % CI)
Age in months (total)			88.3	20.6	20	
Range: 30–119 months						
Male	160	(50.2)			11	1.23 (0.50–3.06)
Female	159	(49.8)			9	1.00
Height for age Z score			−1.65	1.38		
Male			−1.65^a^	1.31		
Female			−1.65^a^	1.44		
Stunting	137	(42.9)			14	3.34 (1.25–8.93)
No stunting	182	(57.1)			6	1.00
Weight for age Z score			−1.66	1.17		
Male			−1.72^b^	1.10		
Female			−1.60^b^	1.24		
Underweight	123	(38.6)			9	1.33 (0.53–3.30)
No underweight	196	(61.4)			11	1.00
Hemoglobin g/L			119.0	12.6		–
Male			118.9^c^	13.4		–
Female			119.2^c^	11.7		–
59 months or younger	33	(10.3)	117.9^d^	12.4		
60 months or younger	286	(89.7)	119.2^d^	12.6		
Anaemia	92	(28.8)			13	5.17 (1.99–13.43)
No anaemia	227	(71.2)			7	1.00
Male	52	(32.5)				1.11 (0.96–1.27)
Female	40	(24.2)				1.00
Severe	2	(0.6)			0	
Moderate	49	(15.4)			10	
Mild	41	(12.9)			3	

p values by t test

^a^p=0.99

^b^p=0.36

^c^p=0.81

^d^p=0.60

### Key findings

Table 2 shows the results for asymptomatic malaria, anaemia, and growth status. Among the total sample, the OR for being anaemic and having an asymptomatic malaria infection was 5.17 [95 % CI 1.99–13.43]. The relationship remained significant for children underweight (OR 5.35, 95 % CI 1.26–22.74), and not underweight (OR 5.00, 95 % CI 1.40–17.82) and for children stunted (OR 4.18, 95 % CI 1.31–13.34) and not stunting (OR 6.38, 95 % CI 1.13–36.08).

**Table 2:** Asymptomatic malaria, anemia and growth status

	Asymptomatic malaria	Anemia		OR (95 % CI)
		No N (%)	Yes N (%)	
Total sample (N = 319)	+	7 (35.0)	13 (65.0)	5.17 (1.99–13.43)*
	−	220 (73.6)	79 (26.4)	1.00
Underweight/not underweight				
No underweight (n = 196)	+	4 (36.4)	7 (63.6)	5.00 (1.40 − 17.82)*
	−	137 (74.1)	48 (25.9)	1.00
Underweight (n = 123)	+	3 (33.3)	6 (66.7)	5.35 (1.26–22.74)*
	−	83 (72.8)	31 (27.2)	1.00
Stunting/not stunting				
No stunting	+	2 (33.3)	4 (66.7)	6.38 (1.13–36.08)*
	–	134 (76.1)	42 (23.9)	1.00
Stunting	+	5 (35.7)	9 (64.3)	4.18 (1.31–13.34)*
	–	86 (69.9)	37 (30.1)	1.00

* p < 0.05

Table 3 shows the results of the multivariate linear regression analysis. Being underweight was not significant in this model whereas stunting was, indicating that stunting was associated with a decreased Hb level after adjusting for asymptomatic infection.

**Table 3:** Multivariate linear regression analysis on hemoglobin level (g/dL)

Variables	Hemoglobin level		
	Unstandardized coefficient		Standardized coefficient
	B	Std. error	β
Model with underweight			
* Asymptomatic Pf* infection^a,^*	−1.27	0.28	−0.24
Age in months	0.003	0.003	0.04
Underweight^a^	−0.05	0.14	−0.02
Constant*	11.84	0.30	
Model with stunting			
* Asymptomatic Pf* infection^a,^*	−1.19	0.28	−0.23
Age in months	0.003	0.003	0.05
Stunting^a,^*	−0.28	0.14	−0.11
Constant^a^	11.87	0.29	

^a^Inputted as binary variable

* p < 0.05

## Discussion

Among children in villages in Lao PDR, a significant association was indicated between asymptomatic malaria and the risk of anaemia and stunted children were also more likely to have an asymptomatic malaria infection. The results of the multivariate analysis also indicated that asymptomatic malaria correlated with a lower Hb level, and stunting was associated with the anaemia, even after adjusting for asymptomatic infection.

Although the results of present study showed that asymptomatic malaria was significantly associated with stunting, previous studies have presented inconsistent findings for this association [26, 28, 33, 44]. For example, while a previous study conducted in Lao PDR indicated that wasted children were more likely to be infected with malaria [33], in a study of Ghanaian children, stunting was not significantly associated with asymptomatic malaria [26].

Some studies suggest that children’s malnutrition may have protective effects against malaria [28, 44]. Conversely, other studies suggest malnourished children seem to be more susceptible to malaria because of decreased immune system functioning [45]. For example, a reduced immunoglobulin G antibody response to *P. falciparum* was observed in children with malnutrition [46], and the relative risk of morbidity due to malaria infections was higher and more consistent in children with a low body mass index [47]. In addition, a nationally representative survey conducted in Equatorial Guinea indicated that malnutrition had a significant relationship with malaria parasitaemia in children [48]. However, complex interactions between malnutrition and the risk and reaction to malaria infection have been indicated in studies [45, 47, 49]. An approach targeting not only malaria parasite but also the comprehensive health status of host such as immunity level [46] would be necessary.

In the multivariate analysis in this study, the association between stunting and anaemia remained even after adjusting for other factors. However, due to the complex relationship between malaria and anaemia, a comprehensive approach is necessary to understand [34, 49, 50]. To interpret these observations, more information, such as the duration of infection and mean parasitaemia levels, should be included in analyses [17].

Severe anaemia was not found among the participants infected with aymptomatic malaria, as in a study conducted in South Ethiopia [51]. In order to study the longitudinal effect, the severity of anaemia would be important aspect for the future observation. Although malaria has been emphasized as acute disease, studies explore chronical effect of asymptomatic malaria, such as disability-adjusted life years would be expected.

This study has several limitations. First, the cross-sectional nature of the data precluded an analysis of the complex, mutual interaction between malaria infection and malnutrition [45]. This interaction is important because malaria infection leads to compromised growth and a compromised nutritional status that in turn leads to increased susceptibility to malaria infection [52]. Furthermore, malnutrition impairs the function of innate and adaptive immunity, which is important for defense against parasitic infection [53], and repeated malaria infections might lead to anaemia and chronic ill health [3]. Therefore, longitudinal studies are needed to help clarify this issue.

Second, the sample was collected by convenience sampling, and as this study was conducted during dry season, only a small proportion of the participants had an asymptomatic malaria infection. Thus, this might not be representative of other populations.

Third, data on other parasitic diseases than *P. falciparum* were not collected due to limited resources and time to make samples for PCR analysis and Hb level in the field. However, one study has shown that other parasitic diseases, such as hookworm infection, may be stronger predictors of Hb levels than sex, malarial parasitaemia, and *Ascaris lumbricoides* infection [54], while *Plasmodium vivax* infection has also been suggested as a predictor of anaemia [55]. Therefore, future studies should include other parasites to further explain the relationship between asymptomatic malaria and anaemia.

Fourth, children aged 120 months or younger, being asymptomatic on the day of the data collection were eligible for inclusion. Therefore, patients with anaemia caused by a recent malaria episode that had cleared by the day of the data collection [18] may have been included in the analysis.

Finally, registered age in months could not be used due to coordination problems and insufficient memory of caregivers. The date of birth is important information to evaluate the growth of children and this can be solved asking approximate date with the help of event calendar during the interview with caregiver [56]. However, such interviews could not be done due to limited time and other resources. This will limit the validity and generalization of the results of this study. Despite these limitations, this study also has several strengths. For example, although PCR is limited because deoxyribonucleic acid fragments remain in the blood for a short time and observations using the PCR technique would capture only living parasites [57, 58], light microscopy is not able to assess submicroscopic infections [59]. Because submicroscopic malaria infections can contribute to the prevalence of anaemia [59], and submicroscopic *P. falciparum* gametocyte carriers could be an infectious reservoir in areas of seasonal transmission [60, 61], PCR-based methods, rather than light microscopy, are a useful and important tool for the detection of the malarial parasite given their sensitivity to detect an infection [62, 63].

In addition, most of the major studies on asymptomatic malaria have been conducted in African regions while epidemiological information on asymptomatic malaria in Asian countries has been limited [13, 33, 47, 55, 63–68]. Given that anaemia, malnutrition, and malaria are public health concerns in Lao PDR, the results of this study are clinically important for understanding the health status and nutritional problems among children in this country.

## Conclusions

In this study, asymptomatic malaria was shown to be associated with anaemia in children, and stunted children were more likely to have lower Hb levels. However, further studies are necessary to understand the impact of asymptomatic malaria on the health status of children in Lao PDR.

## Abbreviations

CI: confidence interval
Hb: haemoglobin
Lao PDR: Lao People’s Democratic Republic
OR: odds ratio
PCR: polymerase chain reaction
SD: standard deviations
WHO: World Health Organization
